# Nasal Bacterial Microbiome Differs Between Healthy Controls and Those With Asthma and Allergic Rhinitis

**DOI:** 10.3389/fcimb.2022.841995

**Published:** 2022-03-03

**Authors:** Meiping Chen, Shiyi He, Phoebe Miles, Chunlin Li, Yijun Ge, Xuechan Yu, Linfeng Wang, Weina Huang, Xue Kong, Shanni Ma, Yiting Li, Qingwen Jiang, Wen Zhang, Chao Cao

**Affiliations:** ^1^ School of Medicine, Ningbo University, Ningbo, China; ^2^ Department of Respiratory and Critical Care Medicine, Ningbo First Hospital, Ningbo, China; ^3^ Faculty of Humanities and Social Sciences, University of Nottingham Ningbo, Ningbo, China; ^4^ Department of Otorhinolaryngology-Head and Neck Surgery, Ningbo First Hospital, Ningbo, China

**Keywords:** asthma, allergic rhinitis, nasal microbiome, inflammation, disease control

## Abstract

Perturbation of the microbiome has numerous associations with the phenotypes and progression in chronic airways disease. However, the differences in the nasal microbiome in asthma and allergic rhinitis (AR) have not been defined. We examined whether the nasal microbiome would vary among different comorbidities in asthma and AR and that those differences may be associated with the severity of asthma. Nasal lavage fluid was collected from 110 participants, including 20 healthy controls, 30 subjects with AR, 30 subjects with asthma and 30 subjects with combined asthma + AR. The Asthma Control Questionnaire (ACQ-7) was used to evaluate asthma control status. Using 16S rRNA bacterial gene sequencing, we analyzed nasal microbiome in patients with asthma, AR, combined asthma + AR, and healthy controls. Bacterial diversity was analyzed in corresponding with α diversity indices (Chao and Shannon index). Compared with healthy controls, the Chao index tended to be lower in subjects with AR (*P* = 0.001), asthma (*P* = 0.001), and combined asthma + AR (*P* = 0.001) when compared with healthy controls. Furthermore, the Shannon index was significantly lower in subjects with asthma (*P* = 0.013) and comorbid asthma with AR (*P* = 0.004) than the control subjects. Disparity in the structure and composition of nasal bacteria were also observed among the four groups. Furthermore, patients with combined asthma + AR and isolated asthma were divided into two groups according to the level of disease control: partially or well-controlled and uncontrolled asthma. The mean relative abundance observed in the groups mentioned the genera of *Pseudoflavonifractor* were dominated in patients with well and partially controlled disease, in both isolated asthma and combined asthma + AR. In subjects with uncontrolled asthma and combined asthma + AR, a lower evenness and richness (Shannon index, *P* = 0.040) was observed in nasal microbiome composition. Importantly, lower evenness and richness in the nasal microbiome may be associated with poor disease control in combined asthma + AR. This study showed the upper airway microbiome is associated with airway inflammation disorders and the level of asthma control.

## Introduction

Perturbation of the microbiome has numerous associations with the phenotypes and progression in chronic airways disease (Fazlollahi et al., 2018; Mac Aogáin et al., 2020). Asthma and allergic rhinitis (AR) are among the commonest chronic inflammatory respiratory diseases. These diseases are intimately linked to the human microbiome and have received much attention recently (Eguiluz-Gracia et al., 2020; Reijula et al., 2020). Previous studies have documented that the bacterial microbiome of different mucosal surfaces is critically involved in allergic airway inflammation (Barcik et al., 2020; Morin et al., 2020). Many studies showed that dysbiosis of the gut microbiome in early childhood could disrupt normal immunoregulation and potentially influence the development of asthma and allergies (Sbihi et al., 2019; Li et al., 2021; McDonnell et al., 2021).

Although previously considered sterile, the lower respiratory tract harbors complex bacterial communities (Huffnagle et al., 2017). Increasing data suggest that the composition and structure of the bronchial microbiome differ between those with allergic respiratory disease and healthy subjects (Huang et al., 2015; Durack et al., 2020). Bronchoalveolar lavage fluid was enriched with *Rothia*, and *Bacteroides* species, whereas depletion of *Sphingomonas* and *Halomonas* was observed in asthmatic patients, with more eosinophils compared with healthy subjects (Sverrild et al., 2017). In addition, one study indicated that 103 taxa including the genus of *Prevotella*, *Haemophilus*, and *Fusobacterium* enrichments differed significantly between asthma with or without atopy; these two groups shared 26% and 29% bronchial bacteria compared to healthy controls (Durack et al., 2017). Furthermore, the endoscopy-guided swab samples in AR were characterized by enrichment of *Propionibacterium* and *Corynebacterium*, but *Streptococcus* were decreased (Lal et al., 2017). Although the airway microbiome of chronic inflammatory respiratory disease conducted by endoscope has been well studied, it is an invasive investigation for patients, not without risks (Durack et al., 2018).

The nasal passage is colonized with a diverse array of microbes, including bacterial, fungi, and viruses (de Steenhuijsen Piters et al., 2019; Mitsi et al., 2020). Furthermore, the nasal mucosa is the first contact point that is exposed to the external environment. In contrast to measuring the bronchial microbiome by bronchoscopy, assessment of nasal microbiome involves minimally invasive methods (Durack et al., 2018). Therefore, the nasal microbiome becomes another potential source to help researchers understand the interaction between chronic inflammatory disorders and the microbiome. One previous research revealed the mean relative abundance of *Neisseria and Haemophilus* in the anterior nares was approximately 0.24% and 0.46% for healthy controls. While the relative abundance decreased for the above two genera in patients with tissue infections (Johnson et al., 2015). The traditional hypothesis of “one airway, one disease” is routinely used to express the pathogenic link between asthma and AR (Grossman, 1997). However, very few studies have investigated the differences in the nasal microbiome in asthma and AR (Mahdavinia, 2018). A previous study uncovered relatively increased amounts of *Bacteroidetes* and *Proteobacteria* in patients with asthma (Fazlollahi et al., 2018). Still, it is unclear whether those differences remain for isolated AR or asthma comorbid AR patients. The relationship between the nasal microbiome and asthma control also remains unknown.

Accordingly, we hypothesized that nasal microbiome would differ among patients with asthma and AR, and those differences may be associated with disease control in patients with asthma. We performed 16S rRNA bacterial gene sequencing on nasal lavage fluid to compare the nasal microbial composition among patients with AR, asthma, asthma and comorbid AR and healthy controls.

## Materials and Methods

### Participants and Study Design

We recruited 110 participants, including 20 healthy controls, 30 subjects with AR, 30 subjects with asthma and 30 subjects with asthma and comorbid AR. Subjects with asthma were diagnosed based on GINA (Global Initiative for Asthma) clinical criteria. Subjects diagnosed with AR or asthma and comorbid AR were confirmed using the Allergic Rhinitis and its Impact on Asthma (ARIA) guideline (Bousquet et al., 2019). Exclusion criteria were as follows: (1) age less than 18 or greater than 80 years; (2) respiratory infection within 4 weeks; (3) antibiotic use within 3 months; (4) systemic steroid therapy within 5 months; (5) pulmonary disease other than asthma; (5) cancer; (6) currently pregnant or lactating.

Smoking status, comorbidities, relevant medical history, medications, first degree relatives with a history of AR or asthma, and type of allergen were recorded for all participants. The severity of AR was quantified using the total nasal symptom score (TNSS). The Asthma Quality of Life Questionnaire (AQLQ) was used to assess the life quality for patients with asthma (Juniper et al., 1999a). The Asthma Control Questionnaire (ACQ-7) was used to evaluate asthma control status (Barnes et al., 2014). Controlled or partially controlled asthma was defined as ACQ less than 1.5, uncontrolled asthma was defined as ACQ more than 1.5 (Juniper et al., 1999b).

This study was conducted at Ningbo First Hospital with approval from the ethics committee of Ningbo First Hospital (approval 2020-R145). All subjects provided written informed consent before participating in the study.

### Pulmonary Function Testing

All participants performed pulmonary function testing according to the American Thoracic Society (ATS) guidelines (Graham et al., 2019). All measurements were undertaken at least three times using the same spirometer (Jager, MasterScreen, Höchberg, Germany) (Hankinson et al., 1999). Pulmonary function was recorded as a percentage of predicted forced expiratory volume in one second (FEV_1_), forced vital capacity (FVC), maximal mid-expiratory flow (MMEF75/25), and peak expiratory flow (PEF).

### Sample Collection

Nasal lavage was performed according to previously described methods (Hirvonen et al., 1999; Frischer et al., 2000). Participants’ heads were briefly held downwards to avoid fluid entering the nasopharynx and allow the liquid to drip into a sterile basin. The nasal passage was slowly instilled with 10 ml of sterile saline, and the nasal lavage was immediately recovered into 15 ml in sterile tubes and placed on ice. The tubes were frozen and stored at -80°C until analysis.

### Sample Processing and Preparation for Sequencing

Total DNA was extracted from 5ml of nasal lavage fluid using DNeasy PowerWater Kit (Qiagen, Hilden, Germany) following instructions provided by the manufacturer. DNA yield and integrity were identified using a Qubit Fluorometer (Qubit, Invitrogen, USA) and 1% agarose gel electrophoresis, respectively. The V3-V4 regions in bacteria 16S rRNA gene of the nasal lavage fluid were amplified with PCR primers 341F (5’-ACTCCTACGGGAGGCAGCAG-3’) and 806R (5’- GGACTACHVGGGTWTCTAAT-3’). The primers were removed before processing. Subsequently, the PCR amplicons were purified with Agencourt AMPure XP magnetic beads, dissolved in Elution Buffer (pH = 8.0) and labelled. The insert fragment size was estimated with an Agilent 2100 Bioanalyzer (Agilent, Santa Clara, CA, USA), then the sample was sequenced on an Illumina HiSeq2500 platform (Illumina, Inc., San Diego, CA, USA) using the PE300 module. Low quality and ambiguous bases were removed using Cutadapt (V2.6) according to the following: (a) raw reads with average quality less than 20, (b) final length less than 75% of their original sequence, (c) reads with an ambiguous bases (N), (d) reads with low complexity (repeats of length more than 10 bases). Amplicon sequence information in the study is available at the Sequence Read Archive (SRA) under BioProject Accession Number PRJNA793600.

The overlap and paired-end reads were processed by the Fast Length Adjustment of Short reads program (FLASH, v1.2.11) (Magoč and Salzberg, 2011) to obtain sequence data. The amount of raw sequence data was more than 50,000 for each sample. Next, sequences were clustered into operational taxonomic units (OTUs) based on a 97% similarity threshold using UPARSE software (v7.0.1090) (Edgar, 2013). OTU representative sequences were classified by Ribosomal Database Project (RDP) Classifier (RDP release version 11.5; release date, 2017.12.0); 0.8 was set as the minimum confidence threshold (Cole et al., 2014). Singleton OTUs and low abundant OTUs were not included in further analyses, additionally, any OTUs not classified as bacteria were removed. Sequences identified as mitochondria were also eliminated. All samples were processed simultaneously in* *the* *same research laboratory (BGI, Shenzhen, China) to control batch variation.

### Bioinformatic Analysis

R (v3.2.1) was applied to determine α-diversity (the evenness and richness of bacteria taxonomic diversity), which was expressed by the Chao, Shannon, and Simpson index. β-diversity (distance between samples, based on the difference in OUT in each sample) was performed by Quantitative Insights into Microbial Ecology (QIME, v1.80) (Caporaso et al., 2010); it was evaluated by the principal coordinates analysis (PCoA) and the Partial least-squares discrimination analysis (PLS-DA). PLS-DA was conducted with R package mixOmics (Rohart et al., 2017). False discovery rate (FDR) was calculated according to the Benjamini-Hochberg correction. The heatmap was plotted with Graph Pad Prism 8 (Graph Pad Software, USA). Linear discriminant analysis of effect size (LEfSe) combined with linear discriminant analysis (LDA) was adopted to explore the differences in taxonomic composition (Segata et al., 2011); LDA > 3.6 was presented in this study (default: 2.0).

### Functional Analysis of Nasal Microbiome

The function of the nasal microbiome was analyzed *via* Phylogenetic Investigation of Communities by Reconstruction of Unobserved States (PICRUSt) and further categorized into Kyoto Encyclopedia of Genes and Genomes (KEGG) pathway (Langille et al., 2013). The associations between variables of bacteria load and metabolic function were evaluated by Pearson correlation coefficient (r).

### Statistical Analysis

Basic characteristics were performed using SPSS software (version 21; SPSS, Inc., Chicago, IL, USA). Categorical variables were compared using the chi-squared test or the Fisher exact test. A two-tailed P value less than 0.05 was considered statistically significant.

## Results

### Patient Cohort

**Figure 1:**
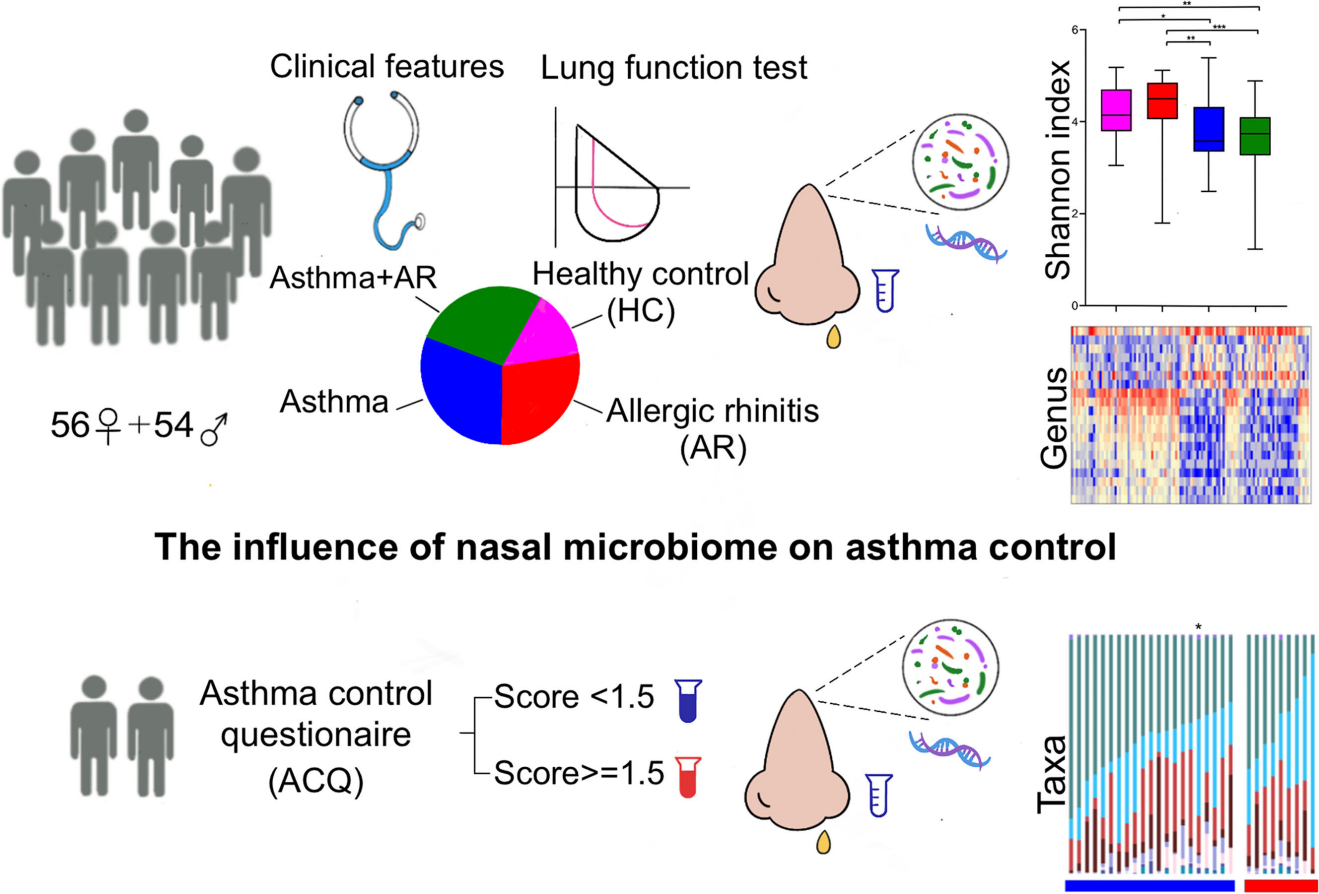
Study design profile. AR, allergic rhinitis; HC, healthy controls; ACQ, Asthma control questionnaire. P < 0.05 was considered as statistically significant, *P < 0.05, **P < 0.01, *** P < 0.001.

A total of 110 participants were* *enrolled, including 90 patients with disease and 20 healthy controls (**Figure 1**). The subjects with disease were divided into three groups, 30 subjects with AR, 30 subjects with asthma and 30 subjects with asthma and comorbid AR. Detailed demographic and baseline characteristics are listed in **Table 1**. No significant differences in age, BMI, sex or smoking status were observed among the groups. Pulmonary function tests were performed for all participants. Family history and allergic of study were listed in **Supplementary Table 1**. Participants The subjects with asthma and those with asthma and comorbid AR were subdivided into groups according to their ACQ score: controlled or partially controlled (defined by ACQ score < 1.5) and uncontrolled (defined by ACQ score > 1.5). The characteristics of the asthmatic patients with or without comorbid AR are summarized in **Supplementary Tables 2, 3**.

**Table 1:** Demographics and clinical characteristics of study participants.

Characteristic	HC	Patients with allergic disease				*P* Value	
		Total	AR	Asthma	Asthma+AR	*P*-HC *vs.* Patients	*P-AR vs. Asthma vs. Asthma + AR*
Subjects (no.)	20	90	30	30	30		
Age (y)	41.00 ± 13.08	39.21 ± 12.19	37.53 ± 10.91	41.23 ± 13.73	38.87 ± 11.90	0.559	0.592
Sex ratio (M/F)	11/9	43/47	14/16	16/14	13/17	0.559	0.732
Smoking status, %						0.208	0.363
Current	3 (15.00)	8 (8.89)	3 (10.00)	3 (10.00)	2 (6.67)		
Ex-smoker	0	11 (12.22)	4 (13.33)	1 (3.33)	6 (20.00)		
Never	17 (85.00)	71 (78.89)	23 (76.67)	26 (86.67)	22 (73.33)		
BMI (kg/m^2^)	24.05 ± 3.02	23.36 ± 3.43	23.60 ± 2.97	22.59 ± 3.35	23.87 ± 3.89	0.406	0.318
FEV_1%_ predicted	102.48 ± 17.18	88.63 ± 20.35	100.72 ± 13.53	83.09 ± 18.50	82.07 ± 22.75	0.006	<0.001
FEV_1_/FVC %	83.38 ± 6.69	80.45 ± 11.17	87.41 ± 6.40	77.30 ± 9.83	76.64 ± 13.03	0.13	<0.001
PEF %	101.12 ± 14.69	89.39 ± 20.71	94.47 ± 10.76	88.72 ± 24.07	84.98 ± 23.95	0.018	0.244
MMEF75/25%	83.40 ± 27.37	70.27 ± 31.81	90.51 ± 21.21	58.15 ± 30.61	61.88 ± 32.77	0.091	<0.001
AQLQ Score	6.99 ± 0.03	—	—	6.50 ± 0.33	6.10 ± 0.55	<0.001^*^	0.002^†^
ACQ Score	0	—	—	1.07 ± 0.78	1.27 ± 0.98	<0.001^*^	0.398^†^

Values are mean ± SD or n (%). HC, healthy controls; D, diseased; AR, allergic rhinitis; Asthma + AR, asthma and comorbid AR. BMI, body mass index; M, male; F, female; FEV1, forced expiratory volume in the first second; FVC, forced vital capacity; PEF, peak expiratory flow; MMEF75/25, maximal mid-expiratory flow; ACQ, the Asthma Control Questionnaire; AQLQ, the Asthma Quality of Life Questionnaire; NA, not applicable. Continuous variables were compared by One Way ANOVA. Categorical variables were compared by Pearson’s chi-square test or Fisher exact test. *Mann-Whitney U test for total patients included asthma and asthma comorbid AR patients compared to HC group (2 group comparison). ^†^student t-test for patients with asthma compared to asthma comorbid AR.

### Nasal Microbiome in Patients With Asthma and Allergic Rhinitis

After filtering for low-quality reads, the average number of clean reads was 61140.73 ± 1538.64 (SD) for subjects for AR, 57586.10 ± 4623.94 for asthma, 55341.50 ± 5747.72 for combined asthma + AR, and 59230.30 ± 6256.84 in the healthy controls, detailed data and species accumulation curves was listed in **Supplement File 1**.

**Figure 2:**
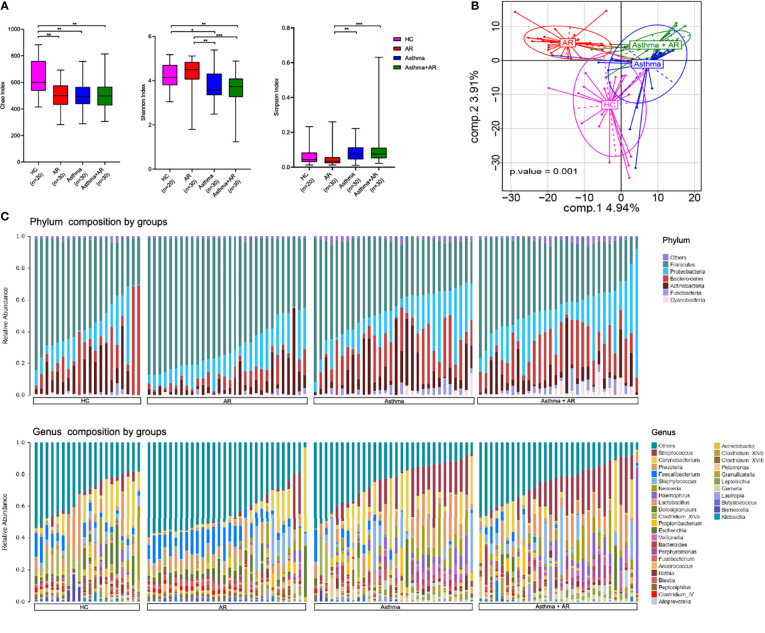
The diversity and composition of the nasal microbiome vary among AR, asthma, combined asthma+AR and healthy controls. **(A)** Box plots of the α-diversity in AR, asthma, combined asthma+AR and healthy controls (Left plot, Chao index; middle plot, Shannon index; right plot, Simpson index). **(B)** Partial least squares discriminant analysis (PLS-DA) representing grouped microbiome profile among AR, asthma, combined asthma+AR and healthy controls. **(C)** Bar plots of the phylum and genus taxonomic levels in AR, asthma, combined asthma+AR and healthy controls. P < 0.05 was considered as statistically significant, *P < 0.05, **P < 0.01, ***P < 0.001.

The researchers used three metrics to compare the α-diversity: the Chao, Shannon, and Simpson index. As shown in **Figure 2A**, the Chao index tended to be lower in subjects with AR (503.38 ± 101.70, *P* = 0.001), asthma (511.01 ± 106.75, *P* = 0.001), and combined asthma + AR (504.99 ± 121.14, *P* = 0.001) when compared with healthy controls (632.26 ± 141.98, **Figure 2A**). Similarly, a lower Shannon index was found in subjects with asthma (3.73 ± 0.68, *P* = 0.013) and combined asthma + AR (3.61 ± 0.75, *P* = 0.004) in comparison to the control subjects (4.21 ± 0l58, **Figure 2A**). However, no significant difference in either the Shannon or Simpson index of nasal microbiome communities was found between healthy controls and patients with AR (**Figure 2A**). Asthma may have a stronger effect on α-diversity than AR in the upper airway.

PLS-DA analyses was applied to study* *the* *structure* *of* *nasal microbiome communities (beta-diversity) among the four groups. Furthermore, PLS-DA showed distinct microbiome composition among all groups (**Figure 2B**). Overall, the results exhibit the specific structure in nasal bacterial community composition among subjects with AR, asthma, combined asthma + AR and healthy controls.

Following initial evaluations for differences in overall nasal bacterial community composition, we investigated the relative abundance of specific taxa in subjects with disease and healthy controls. Evaluation of the general landscape of the nasal microbiome revealed similar bacterial communities at phylum and genus levels (**Figure 2C**). We then evaluated the relative abundance of the bacterial communities at phylum and genus levels within the four groups. *Firmicutes* were the most predominant phylum in all those four groups, followed by *Bacteroidetes*, *Proteobacteria*, *Actinobacteria* (**Figure S1**). Compared with healthy controls, *Firmicutes* were enriched in the samples of subjects with AR (mean relative abundances, 53.55% *vs* 68.73%, FDR adj *P*-value = 0.005) but depleted in subjects with asthma with or without AR (asthma, 53.55% *vs* 45.37%, FDR adj *P*-value < 0.001; Asthma + AR, 53.55% *vs* 41.97%; FDR adj *P*-value < 0.001). No significant difference was found in the relative abundance of *Actinobacteria* or *Proteobacteria* among the four groups with disease (**Figure S2** and **Supplementary Table 4**).

To further confirm these findings, we used the LEfSe analysis (Segata et al., 2011); this found marked differences in the nasal bacteria community among the four groups (**Figure S3**). Subjects with asthma had a high proportion of *Actinomycetaceae*, *Listeriaceae*, and *Neisseriaceae* at the family level. The samples of subjects with combined asthma + AR were enriched with *Provotellaceae*, *Sphingobacteriaceae*, *Rhodocyclaceae*, *Aeromonadaceae*, and *Leptotrichiaceae* (LAD > 2.0, *P* < 0.05, **Figure S3A**). At the genus level, the top 10 most abundant nasal microbiome were selected for further analysis. We identified seven taxa that differed among the four groups: *Streptococcus*, *Prevotella*, *Faecalibacterium*, *Neisseria*, *Lactobacillus, Haemophilus* and *Clostridium_XlVa* (FDR adj *P*-value < 0.05 in each analysis, **Figure S4** and **Supplementary Table 5**). The relative abundance of *Streptococcus* was higher in the asthma (*P* < 0.01) and combined asthma + AR (P < 0.01) groups compared with healthy controls, respectively. However, the relative abundance of *Faecalibacterium*, *Lactobacillus*, and *Clostridium_XlVa* were lower in the asthma and combined asthma + AR groups in relation to healthy controls (*P* < 0.05).

LDA analyses were adopted to determine the specific bacterial taxa in each group (LDA > 3.6, *P* < 0.05; **Figure S3B**). The genera of *Prevotella* and *Kineococcus* characterized the samples of the healthy control subjects. Additionally, 13 genera of the nasal microbiome were differentially abundant in subjects with AR, including *Faecalibacterium*, *Lactobacillus*, *Escherichia* and *Clostridium_IV* genera; 84.6% (11/13) belong to the *Firmicutes* phylum (**Figure S3B**). The genera of *Neisseria* and *Rothia* dominated the microbiome of subjects with asthma; whereas, the genera of *Pelomonas*, *Alloprevotella*, *Leptotrichia*, and *Granulicatella* dominated the microbiome of subjects with asthma and comorbid AR (**Figure S3B**). We used heatmaps to visualize the relative abundance of the dominant taxonomic communities at the genera level of the nasal microbiome in different groups. A higher quantity corresponds to a deeper red color. The heatmap displayed a similar abundance of bacteria in subjects with asthma relative to those with asthma and comorbid AR (**Figure S5**).

**Figure 3:**
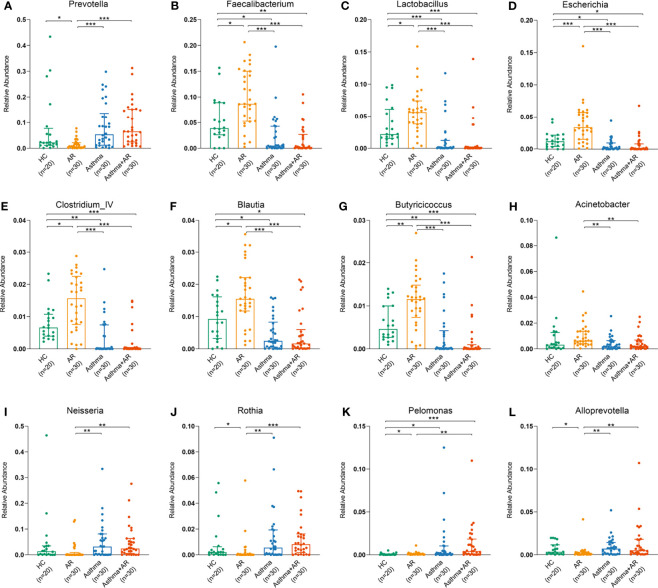
Relative abundance of dominant bacterial genera among AR, asthma, combined asthma+AR and healthy controls. **(A)** *Prevotella*, **(B)** *Faecalibacterium*, **(C)** *Lactobacillus*, **(D)** *Escherichia*, **(E)** *Clostridium_IV*, **(F)** *Blautia*, **(G)** *Butyricicoccus*, **(H)** *Acinetobacter*, **(I)** *Neisseria*, **(J)** *Rothia*, **(K)** Pelomonas, and **(L)** Alloprevotella. Statistical significance was tested by Kruskal-Wallis test with Benjamini-Hochberg procedure. *P* < 0.05 was considered as statistically significant, *P< 0.05, **P < 0.01, ***P < 0.001.

At the genus level, there was no significant difference in taxonomic distribution between the subjects with asthma and those with asthma and comorbid AR (**Figure 3**). However, a significant difference was found in the genera composition between the samples of subjects with AR, asthma, combined asthma + AR and healthy controls (**Figure 3**). The relative abundance [median (interquartile range, IQR)] of *Prevotella* was lower in the AR group compared with healthy controls [0.78% (1.99%) *vs.* 2.23% (6.58%), FDR adj *P*-value = 0.016, **Figure 3A**]. Conversely, the relative abundance of *Faecalibacterium* was greater in the AR group [8.67% (9.71%)] relative to healthy controls [3.91% (6.25%), FDR adj *P*-value = 0.011], subjects with asthma [0.50% (4.20%), FDR adj *P*-value < 0.001], and subjects with asthma and comorbid AR [0.24% (2.66%), FDR adj *P*-value < 0.001, **Figure 3B**). Similarly, the genera of *Lactobacillus*, *Escherichia*, *Clostridium_IV*, *Blautia* and *Butyricicoccus* in the AR group had the highest relative abundance of all groups (**Figure 3C-G**). In contrast, a high level of *Neisseria*, *Rothiawas*, *Pelomonas*, and *Alloprevotella* was detected in subjects with asthma and combined asthma + AR when compared to the healthy controls (**Figure 3I-L**). However, the most of these genera have very low relative abundance across all samples.

**Figure 4:**
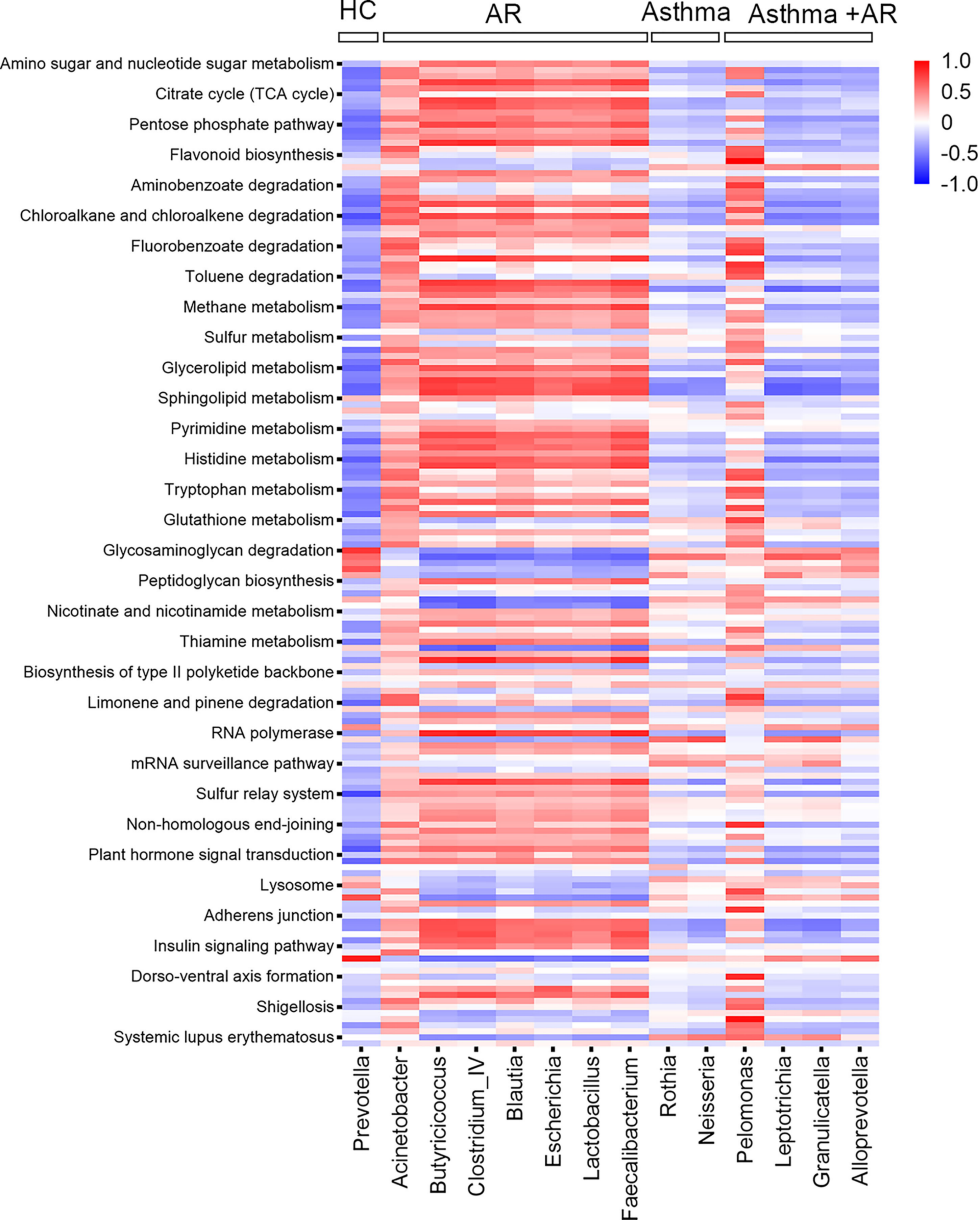
Pearson correction heat maps of predicted KEGG orthologs (KOs) and nasal bacterial taxa among AR, asthma, combined asthma+AR and healthy controls. Blue indicates negative correlations, and red indicates positive correlations. AR, allergic rhinitis; HC, healthy controls.

We further assessed whether the predicted function of the nasal bacterial dominant taxa in different groups differed distribution across the groups. In total, 161 metabolites were analyzed by KEGG pathway analysis. The Pearson correlation heat map in **Figure 4** summarized the clustering of enriched functions among the three groups with disease and the healthy control group. Nasal bacterial communities enriched in AR had multiple functional features related to the pathway of pentose phosphate and galactose metabolism. Whereas, decreased activities in the pathway of primary bile acid biosynthesis were observed in the genus of *Rothia* (Pearson r = - 0.48; *P <*0.001, **Figure 4**) and *Neisseria* (r = - 0.44; *P <*0.001, **Figure 4**), which enriched in subjects with asthma (**Figure 4**). The genera of *Pelomonas* were highly abundant in patients with combined asthma + AR; it was involved in the pathway of lipid metabolism, especially with upregulated fatty acid degradation (Pearson r = 0.62; *P <*0.01, **Figure 4**). Additionally, the genera of *Pelomonas* associated with the metabolism of glutathione (r = 0.726; *P <*0.05, **Figure 4**).

### Nasal Microbiome of Subjects With Asthma According to the Level of Disease Control

**Figure 5:**
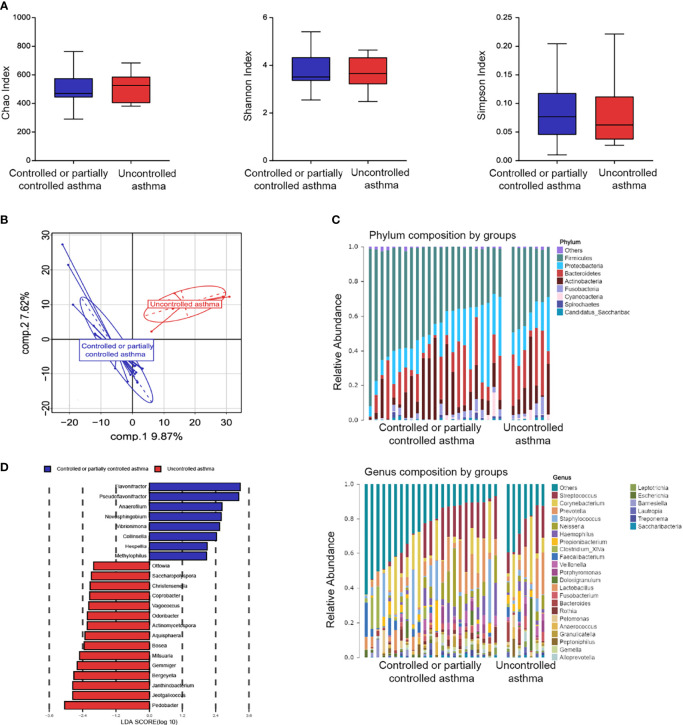
Nasal microbiome composition in asthma according to disease control. **(A)** Box plots of the α-diversity in controlled or partially controlled asthma and uncontrolled asthma (Left plot, Chao index; middle plot, Shannon index; right plot, Simpson index). **(B)** β-diversity based on PLS-DA analysis in controlled or partially controlled asthma and Uncontrolled asthma. **(C)** Bar plots of the phylum and genus taxonomic levels in asthma based on asthma control. **(D)** Taxonomic Cladogram from LEfSe. Taxonomic distribution of nasal microbiome of each group at the different taxon (LDA Score > 2.0).

To further investigate the relationship between nasal microbiome composition and disease control, we divided subjects with asthma into two groups: 23 (76.7%) of them with partially or well-controlled, and 7 (23.3%) subjects with uncontrolled asthma. No significant differences in microbiome evenness or diversities were found according to the Shannon, Chao, and Simpson indexes (**Figure 5A**). β-diversity, based on the PLS-DA analyses, was performed to compare microbial structure. It is notable that there was a clear separation between the two groups from the PLS-DA analysis (**Figure 5B**).

The general features of the nasal microbiome at phylum and genus levels are shown in **Figure 5C**. Five dominant phyla were found in nasal lavage fluid samples: *Firmicutes*, *Proteobacteria*, *Actinobacteria*, *Bacteroidetes* and *Fusobacteria* (**Supplementary Figure 6**). Similarity in the abundance at phylum and genus levels was observed between the two groups (**Supplementary Table 6**). However, a higher proportion of *Cyanobacteria* was found in subjects with uncontrolled asthma, compared to subjects with controlled or partially controlled asthma (**Supplementary Table 6**). In contrast, a lower proportion of *Firmicutes* was observed in subjects with uncontrolled asthma (**Supplementary Table 6**). The LDA analyses were performed to validate the specific genera microbiome according to level of asthma control (LDA > 2.0, P < 0.05, **Figure 5D**). The three top genera of nasal taxa in patients with controlled or partially controlled asthma were *Flavonifractor* (phylum *Firmicutes*), *Pseudoflavonifractor* (phylum *Firmicutes*) and *Anaerofilum* (phylum *Firmicutes*). While *Pedobacter* (phylum *Bacteroidetes*), *Jeotgalicoccus* (phylum *Firmicutes*), and *Janthinobacterium* (phylum *Proteobacteria*) were enriched in the samples of the uncontrolled asthma group (**Figure 5D**).

It was speculated that the bacterial genus enrichment in the upper airway might lead to metabolic changes in subjects with asthma. Here, metabolomics analysis was conducted on nasal bacteria with respect to the enrichment in different degrees of asthma severity. The data presented above suggest that patients with uncontrolled asthma are enriched with the genus of *Janthinobacterium* and *Pedobacter.* Conversely, the genus of *Collinsella, Anaerofilum, Novosphingobium, Flavonifractor, Pseudoflavonifractor*, and *Vibrionimonas* were enriched in patients with controlled or partially controlled asthma. Both the Lipoic acid metabolism (r = 0.47, *P* =0.001) and Vibrio cholerae infection (r = 0.40, *P* = 0.004) pathways exhibited the positive correlation with the bacterial loading of *Janthinobacterium* in subjects with uncontrolled asthma (**Figure S7**). Interestingly, a negative correlation was observed between the pathway of Lipoic acid metabolism and the genera of *Flavonifractor* (r = - 0.53, P < 0.001), which was enriched in patients with controlled and partially controlled asthma.

### Nasal Microbiome in Subjects With Combined Asthma + AR According to the Level of Disease Control

**Figure 6:**
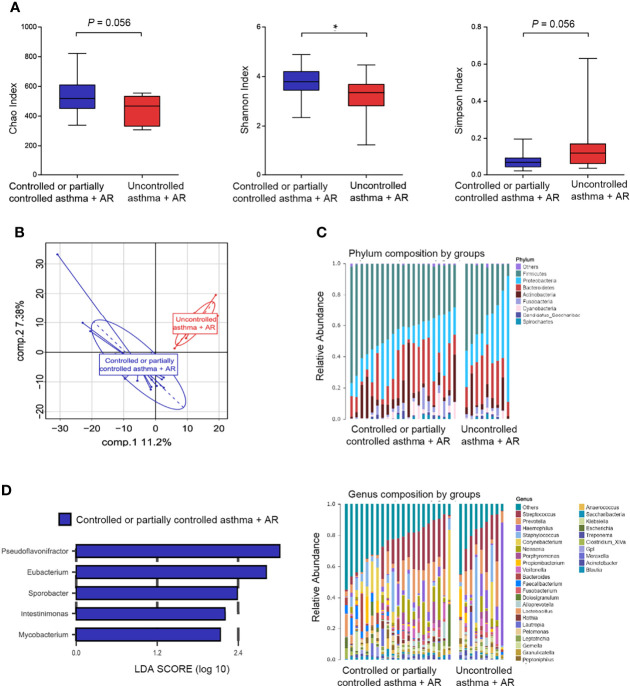
Nasal microbiome composition in combined asthma+AR according to disease control. **(A)** Box plots of the α-diversity in combined asthma+AR group based on different disease control (Left plot, Chao index; middle plot, Shannon index; right plot, Simpson index). **(B)** β-diversity based on PLS-DA analysis in combined asthma+AR according to disease control. **(C)** Bar plots of the phylum and genus taxonomic levels in combined asthma+AR group based on asthma control. **(D)** LDA score computed for genera significantly abundance in combined asthma+AR group based on asthma control (LDA Score > 2.0). P < 0.05 was considered as statistically significant, *P < 0.05.

Among 9 (30%) subjects with uncontrolled asthma and combined asthma + AR, a lower Shannon index (*P* = 0.040) was observed in nasal microbiome composition. This suggests reduced evenness and diversity of nasal bacteria relative to those 21 (70%) subjects with controlled or partially controlled combined asthma + AR. A similar trend was observed in the Chao and Simpson indexes, although the difference was not statistically significant (**Figure 6A**). To determine variation between different asthma control groups in participants with asthma and comorbid AR, PLS-DA analyses were performed. When compared with the uncontrolled asthma and comorbid AR groups, significant separation of PLS-DA was also found between these two groups (**Figure 6B**).

We next assessed the landscape of the nasal microbiome at the phylum and genus levels in all subjects with asthma and comorbid AR (**Figure 6C**). *Proteobacteria* were enriched in the samples of subjects with uncontrolled asthma and comorbid AR; however, the difference was not statistically significant after being adjusted for FDR (**Figure S8** and **Supplementary Table 9**). In nasal lavage samples, the relative abundance of five genera: *Pseudoflavonifractor* (phylum *Firmicutes*), *Eubacterium* (phylum *Firmicutes*), *Sporobacter* (phylum *Firmicutes*), *Intestinimonas* (phylum *Firmicutes*), and *Mycobacterium* (phylum *Actinobacteria*) were enriched in the samples of subjects with controlled or partially controlled in asthma and comorbid AR group (**Figure 6D**).

We further conducted function analysis to explore nasal bacteria in the development of allergic inflammation. The genera of *Eubacterium, Pseudoflavonifractor*, and *Sporobacter* were enriched in patients with controlled or partially controlled combined asthma + AR; they were adapted to assess the correlation between taxa loading and metabolic pathway. The load of *Eubacterium* is closely related to the active pathway of NOD-like receptor signaling (r =0.63, *P* < 0.001) and RNA polymerase (r=0.66, *P* < 0.001) (**Figure S9**).

## Discussion

Microbiome composition and function have been identified as contributing factors in the pathogenesis of inflammation (Sanders et al., 2021). Asthma and AR can be characterized by Th2-dominated airway inflammation (Pinart et al., 2014). However, little is known about the differences in microecology of the upper respiratory tract expression in patients with asthma and AR. Considering that microbiota population and composition host-microbe interactions play an important role in inflammation, we conducted this study to investigate the nasal microbiome of patients with asthma and AR. To the best of our knowledge, this study represents the first and the most comprehensive analysis of the effect of the nasal microbiome in disease control in patients with asthma, with or without AR.

The findings from this study show significant disparities in the structure and composition of nasal bacteria composition between healthy controls and patients with asthma patients with or without comorbid AR. Alpha diversity metrics suggested that bacterial diversity is significantly decreased in patients with asthma and AR versus healthy controls. The nasal microbiome in samples of healthy controls was dominated by six phyla: *Firmicutes, Bacteroidetes, Proteobacteria, Actinobacteria, Cyanobacteria, and Fusobacteria.* The samples of patients with combined asthma + AR were enriched with *Fusobacteria* and *Cyanobacteria*, while the level of *Firmicutes* was depleted. *Firmicutes is one of the most abundant bacteria at the phylum* levels in healthy adults (Eckburg et al., 2005). and it has a critical role in keeping barrier function associated with the pathway of short-chain fatty acids (SCFA) and secondary bile acids (BAs) (Sartor and Wu, 2017). Decreased level of *Firmicutes* was found in the sputum samples in patients with mild active asthma (Marri et al., 2013). Another study revealed that the proportions of *Proteobacteria/Firmicutes* associated with asthma exacerbation (Ghebre et al., 2018). Therefore, the changes in the *Firmicutes* may provide insight into the allergic disease. However, more research and data are needed in support of future clinical applications.

Multiple studies support the potential roles of the microbiome in allergic pathogenesis and disease course (Tsilochristou et al., 2019; Barcik et al., 2020; Morin et al., 2020). The upper and lower airway bacterial community composition and diversity were inconsistent. A study showed a similar bacterial alpha-diversity in sputum in patients with asthma and healthy controls, but a decreased sputum bacterial alpha-diversity was observed in patients with elevated levels of pro-inflammatory cytokines than those with lower levels of pro-inflammatory cytokines (Durack et al., 2020). Furthermore, subjects with asthma were uniquely enriched bronchial bacterial in the genus of *Neisseria, Haemophilus*, and *Fusobacteriumm* (Durack et al., 2017). However, the genus of *Moraxella* and *Staphylococcus* were most frequently detected in the upper airway microbiome in patients with asthma, and the bacterial variance in nasal airway microbial composition was related to asthma exacerbation in pediatric patients (McCauley et al., 2019). Our findings demonstrate the link between microbiome alterations of the upper respiratory tract and asthma control. *Cyanobacteria*, a phylum of Gram-negative bacteria, was found at relatively higher proportions in the samples of subjects with uncontrolled asthma. The phylum of *Cyanobacteria*, a Gram-negative bacterial lipopolysaccharide/s (LPS), is attributed to host-mediated responses and may result in allergic responses (Lee et al., 2021). Moreover, a higher amount of *Janthinobacterium* was significantly associated with uncontrolled asthma in this study. Existing research in infants (0-6 months) showed that an increase in the abundance of *Janthinobacterium* might be linked to the development of respiratory tract infections (Man et al., 2019). Additional evidence has also shown that respiratory tract infections were the leading cause of asthma exacerbations (Hansel et al., 2013). Our study demonstrated that the genera of *Pedobacter* and *Jeotgalicoccus* were dominant in the samples of subjects with uncontrolled asthma. We sought to identify and establish a link between the nasal microbiome and asthma control in subjects with established disease. High proportions of *Cyanobacteria, Pedobacter*, *Jeotgalicoccus*, and *Janthinobacterium* were associated with poor asthma control. This analysis may help improve the understanding of underlying pathobiology and potential biomarkers and provide new therapeutic strategies for asthma treatment (Chung, 2017).

It is well established that altered bacterial diversity increases the risk of immune-mediated diseases (Bisgaard et al., 2007; Teo et al., 2015). We found a lower α-diversity in the samples of subjects with uncontrolled combined asthma + AR. Decreased bacterial diversity was found in the nasal microbiome of subjects with uncontrolled asthma and comorbid AR, but not in subjects with uncontrolled asthma. This finding is likely attributable to the association between persistent allergic rhinitis and uncontrolled asthma by enhancing lower respiratory tract inflammation (Oka et al., 2014). Our results showed that the genera of *Pseudoflavonifractor, Eubacterium*, and *Sporobacter* were characterized in the samples of subjects with asthma and comorbid AR; these genera all belong to the *Firmicutes* phylum. Our findings suggest that the genus of *Pseudoflavonifractor* were beneficial for asthma control in asthma with or without comorbid AR. *Pseudoflavonifractor* produces butyrate, which is associated with immune-modulation (Alam et al., 2021). It’s possible that the butyrate attenuated eosinophil trafficking and survival to protect against allergic airway inflammation (Theiler et al., 2019).

It is noteworthy that our research found a dissimilar bacterial composition in the samples of subjects with asthma and combined asthma + AR relative to the samples of healthy controls. At the phylum level, samples of the subjects with combined asthma + AR had a higher relative abundance of *Fusobacteria* and *Cyanobacteria*. Additionally, the subjects with asthma and comorbid AR were enriched with the genus of *Haemophilus.* However, the differences in this bacterial microbiome were not statistically significant in subjects with asthma compared to healthy controls. *Haemophilus* was detected in the respiratory tract with low abundance based on the Human Microbiome Project, and the relative abundance was about 1% on average (Zhou et al., 2013). Our study showed that the mean relative abundance of *Haemophilus* was 6.13% and 1.42% in asthma and comorbid AR and healthy controls, respectively. Enrichment of *Haemophilus* within the upper airway microbiome may be associated with asthma and appears to be a risk factor for the development of asthma (Durack et al., 2017). Furthermore, the genus of *Pelomonas* was enriched in patients with combined asthma + AR; it has a highly positive correction with the pathway of fatty acid degradation. Metabolomic profiling exhibited the inverse relationship between polyunsaturated fatty acids (PUFAs) and allergic disease (Lee-Sarwar et al., 2019a; Lee-Sarwar et al., 2019b). These results support that regulation between microbiome alterations may potentially result in autoimmune disease or allergies.

This study provided an improved understanding of airway pathogens and allergic airway inflammation. We found that alterations of the upper respiratory microbiome may contribute to multiple allergic diseases. In particular, a different proportion of *Prevotella*, *Feacalibacterium*, *Lactobacillus*, *Escherichia*, *Neisseria and Haemophilus* may result in various types of allergic respiratory disease, such as AR, asthma, or combined asthma + AR. Furthermore, the nasal microbiome had an impact on the level of asthma control. *Pseudoflavonifractor* may be beneficial for asthma control. However, *Cyanobacteria*, *Pedobacter*, *Jeotgalicoccus*, and *Janthinobacterium* may contribute to poor asthma control. Moreover, the distribution of airway microbes in patients with asthma may be shifted by medicine. A previous study showed that when compared to placebo treatment, relative abundance of *Microbacteriaceae*, *Neisseria* and *Moraxella* increased, and *Fusobacterium* decreased in the group receiving inhaled corticosteroid treatment (ICS) (Durack et al., 2017). Additionally, another research indicated that use of Azithromycin reduced the levels of *Haemophilus* influenzae load in patients with asthma (Taylor et al., 2019). Antibiotics can affect chronic inflammation *via* multiple pathways, which makes it difficult to identify the immune mechanism (Taylor et al., 2019).

Some limitations of this study need to be addressed. First, we cannot completely rule out that medicine may influence the respiratory bacterial composition, although all subjects included had no history of antibiotic use or systemic steroid therapy within 3 months. Second, the study did not measure bacterial loading, but the relative abundance of the bacteria may also reflect the microbial differences to a certain extent. Third, we recognize that the data of immune markers related to allergic inflammation was missed. We will incorporate flow cytometry for immune markers to provide further evidence for changes in bacterial community composition and diversity in the next study. Moreover, DNA extraction controls were not included in this study. However, nasal lavage fluid collection was conducted by two trained team members that strictly followed the principles of sterility. It is possible that the significant changes in very low abundant taxa may be due to contamination. Furthermore, potential genetic susceptibility for an allergen may influence the structures of the bacterial communities, thus the history of first degree relatives suffering from asthma/allergic rhinitis should be considered in the futures. Therefore, we must acknowledged have not proven that the nasal microbiome had an impact on the level of asthma control. However, we showed some taxa were associated with level of asthma control. Further studies regarding the alteration bacterial taxa and allergic inflammation in animal models are warranted.

In summary, decreased bacterial diversity was found at different degrees in subjects with AR, asthma and combined asthma + AR. This study also presented the disparities in structure and composition of nasal bacteria in patients with respiratory disease. Lower evenness and richness in the nasal microbiome may increase the risk of poor asthma control in patients with asthma and comorbid AR. At the same time, the genera of *Pseudoflavonifractor* (phylum *Firmicutes*) dominated the microbiome of both controlled or partially controlled groups in isolated asthma and combined asthma + AR. The finding from this may help clinicians have a better understanding of airway microbiome and allergic airway inflammation.

## Data Availability Statement

The datasets presented in this study can be found in online repositories. The names of the repository and accession number can be found below: https://www.ncbi.nlm.nih.gov/, PRJNA793600.

## Ethics Statement

The studies involving human participants were reviewed and approved by the ethics committee of Ningbo First Hospital (approval 2020-R145). The patients/participants provided their written informed consent to participate in this study.

## Author Contributions

CC designed research and participated in manuscript writing. MC, SH, PM, CL, YG, XY, LW, WH, XK, SM, YL, QJ and WZ conducted sampling and clinical measures, carried out the experiments, analyzed data, and drafted the manuscript. All authors contributed to the article and approved the submitted version.

## Funding

This study was supported by the National Natural Science Foundation of China (82170016). The funders of the study had no role in study design, data collection, data analysis, data interpretation, or writing of the manuscript.

## Conflict of Interest

The authors declare that the research was conducted in the absence of any commercial or financial relationships that could be construed as a potential conflict of interest.

## Publisher’s Note

All claims expressed in this article are solely those of the authors and do not necessarily represent those of their affiliated organizations, or those of the publisher, the editors and the reviewers. Any product that may be evaluated in this article, or claim that may be made by its manufacturer, is not guaranteed or endorsed by the publisher.
