# Supplementary material for: Nasal Bacterial Microbiome Differs Between Healthy Controls and Those With Asthma and Allergic Rhinitis
Source: Front Cell Infect Microbiol. 2022 Mar 3;12:841995. doi: 10.3389/fcimb.2022.841995 (PMC8928226; doi:10.3389/fcimb.2022.841995)
Supplement: Supplementary file 1 [file Table_1.pdf]

Supplemental Information

Nasal Bacterial Microbiome Differs Between Healthy Controls and those with Asthma and Allergic Rhinitis

Running title: Asthma and AR Microbiome Differs

Meiping Chen, Shiyi He, Phoebe Miles, Chunlin Li, Yijun Ge, Xuechan Yu, Linfeng Wang, Weina Huang, Xue Kong, Shanni Ma, Yiting Li, Qingwen jiang, Wen Zhang, Chao Cao

SUPPLEMENTARY FIGURES

Supplementary Figure 1: Relative taxon abundance in subjects with AR, asthma, asthma and comorbid AR, and healthy controls.

Supplementary Figure 2: Taxonomic distribution of top 6 major abundant bacterial phyla in different allergic groups. (A) *Firmicutes*, (B) *Bacteroidetes*, (C) *Proteobacteria,* (D) *Actinobacteria,* (E) *Cyanobacteria,* (F) *Fusobacteria.* Statistical significance was tested by Kruskal-Wallis test with Benjamini-Hochberg procedure. $P < 0.05$ was considered as statistically significant, *$P < 0.05$, **$P < 0.01$, *** $P < 0.001$.

Supplementary Figure 3: The diversity and composition of the nasal microbiome vary among AR, asthma, combined asthma+AR and healthy controls.

(A) Taxonomic Cladogram from LEfSe, taxonomic distribution of significant taxa among nasal microbiome communities from AR, asthma, combined asthma + AR and healthy controls. Each node corresponds to a taxonomic type. The yellow nodes represent the taxonomic no statistical significance (linear discriminant analysis [LDA] Score > 2.0).

(B) LDA score computed for genera significantly abundance in controlled or partially controlled asthma and Uncontrolled asthma (LDA Score > 2.0).

Supplementary Figure 4: Taxonomic distribution of top 10 major abundant bacterial genera in different allergic groups. (A) *Streptococcus*, (B) *Corynebacterium*, (C) *Prevotella*, (D) *Faecalibacterium*, (E) *Staphylococcus*, (F) *Neisseria*, (G) *Lactobacillus,* (H) *Haemophilus*, (I) *Dolosigranulum* and (J) *Clostridium_XlVa.* Statistical significance was tested by Kruskal-Wallis test with Benjamini-Hochberg procedure. $P < 0.05$ was considered as statistically significant, *$P < 0.05$, **$P < 0.01$, *** $P < 0.001$.

Supplementary Figure 5: Heatmap of dominant taxonomic communities at the genera level. Higher abundance corresponds to a red color. Less abundance corresponds to a blue color.

Supplementary Figure 6: Relative taxon abundance comparisons in asthma according to disease control. A and B representing the relative abundance at phylum and genus levels, respectively.

Supplementary Figure 7: Pearson correction heat maps of predicted KEGG orthologs (KOs) and nasal bacterial taxa in asthma according to disease control. Blue indicates negative correlations, and red indicates positive correlations.

Supplementary Figure 8: Relative taxon abundance comparisons in combined asthma + AR according to disease control. A and B representing the relative abundance at phylum and genus levels, respectively.

Supplementary Figure 9: Pearson correction heat maps of predicted KEGG orthologs (KOs) and nasal bacterial taxa in in combined asthma + AR according to disease control. Blue indicates negative correlations, and red indicates positive correlations. AR, allergic rhinitis.

Supplementary Figure 1

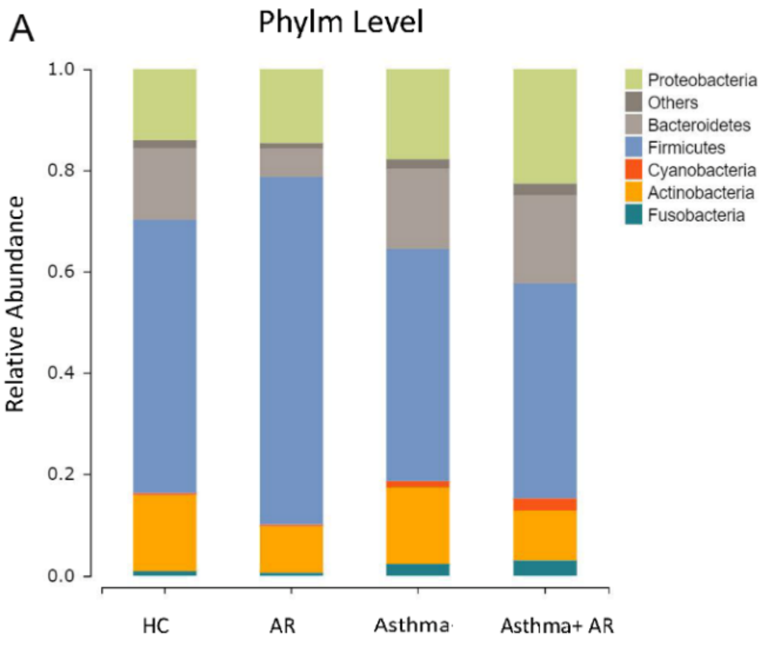

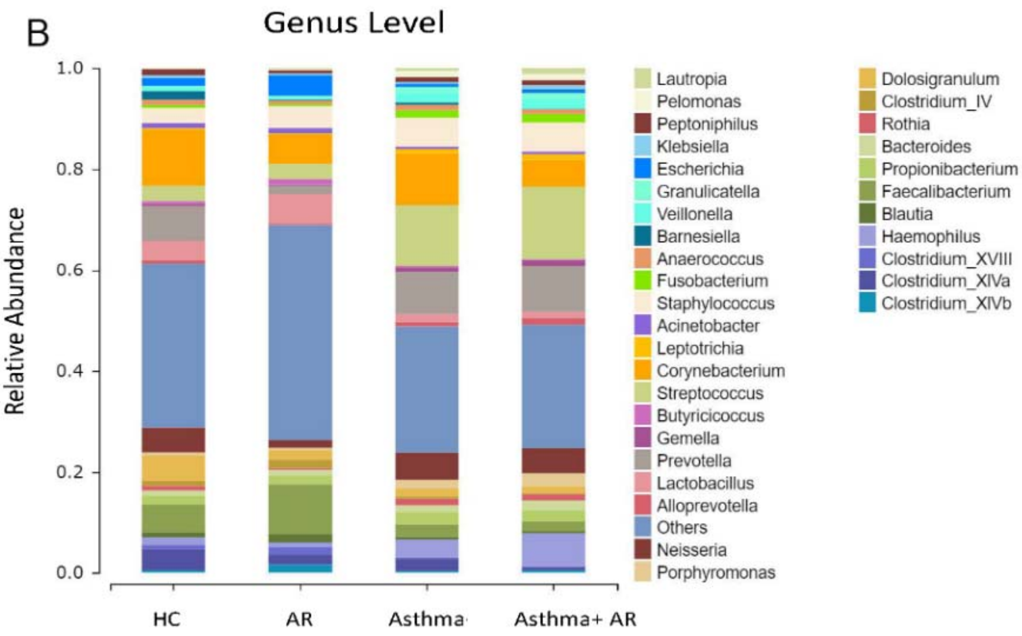

Supplementary Figure 2

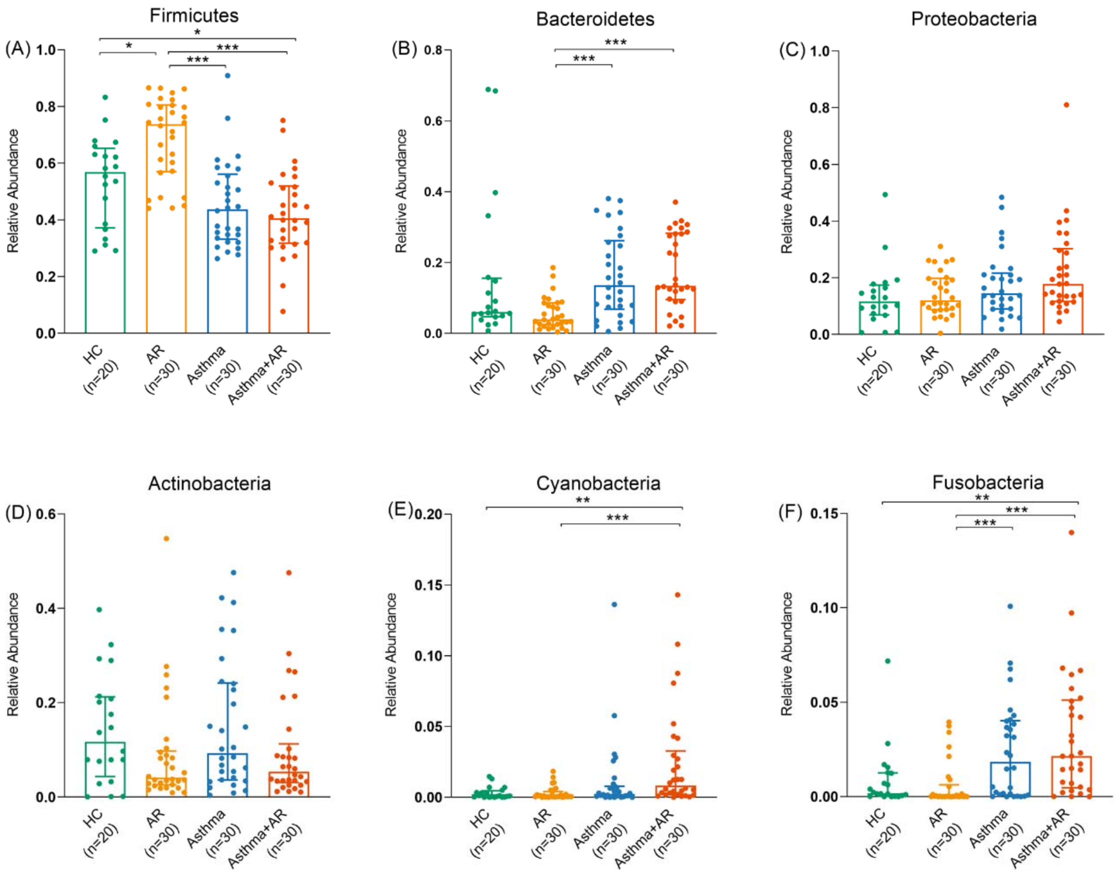

Supplementary Figure 3

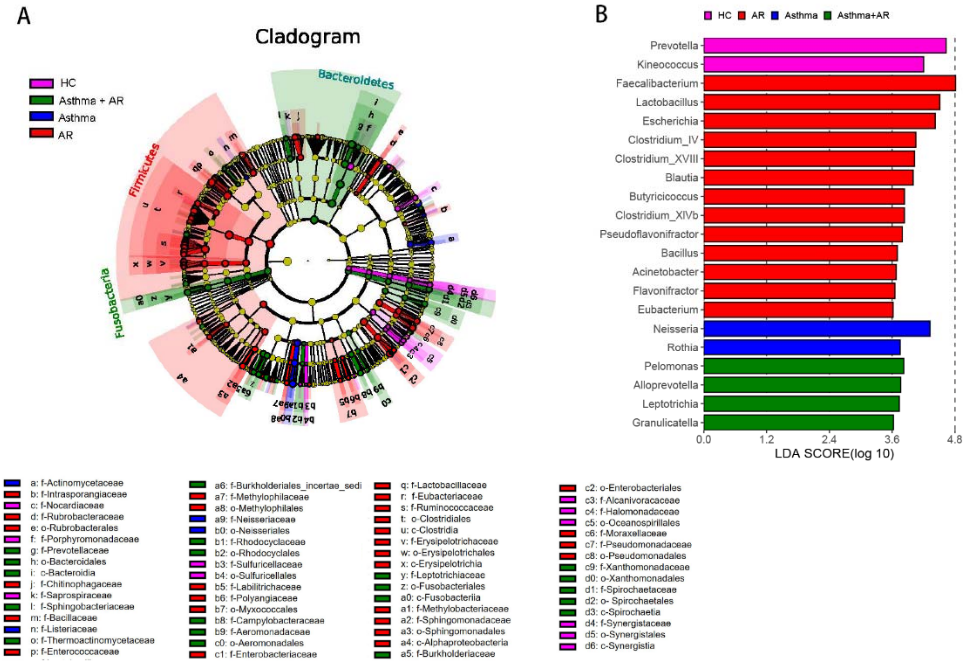

Supplementary Figure 4

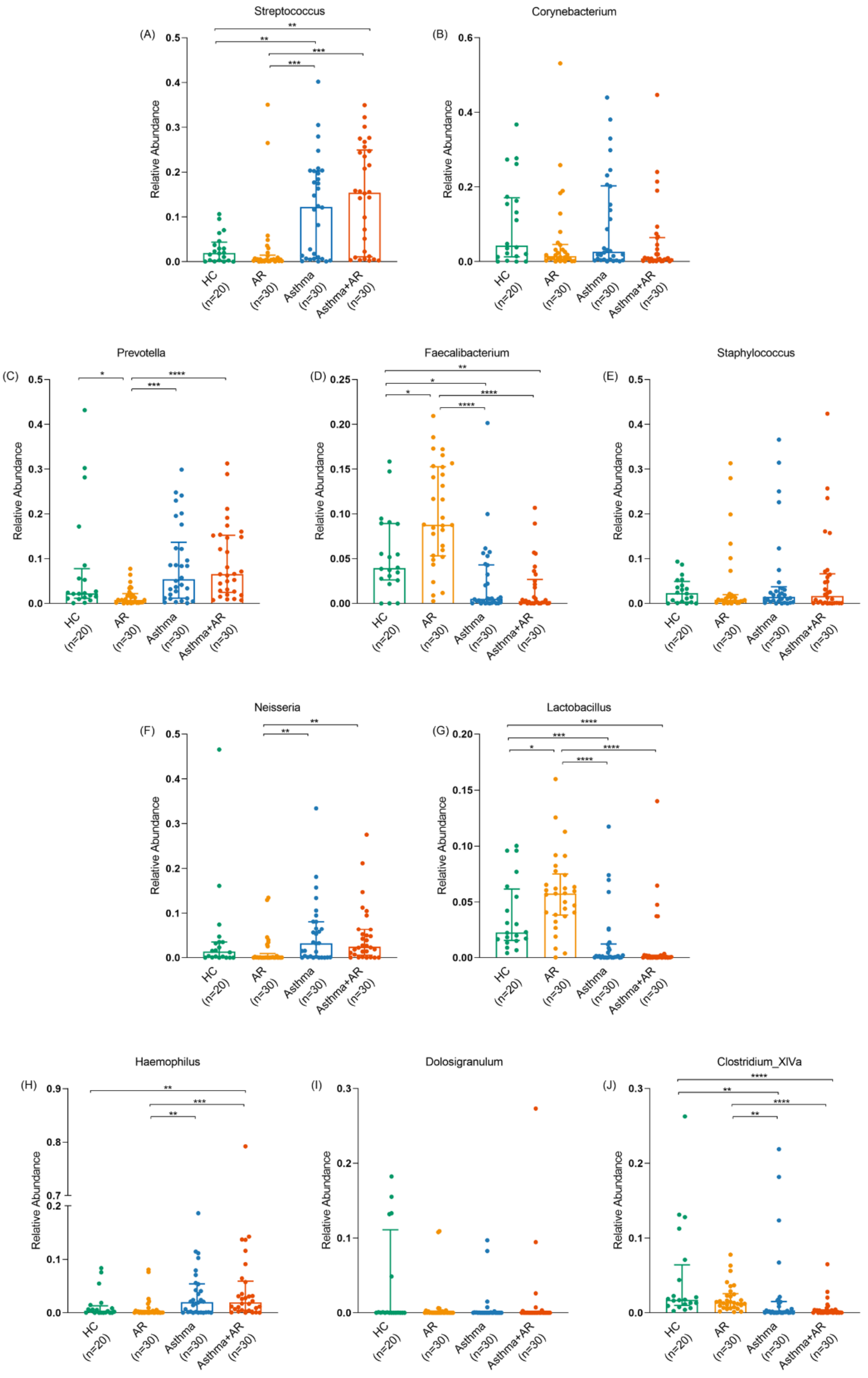

Supplementary Figure 5

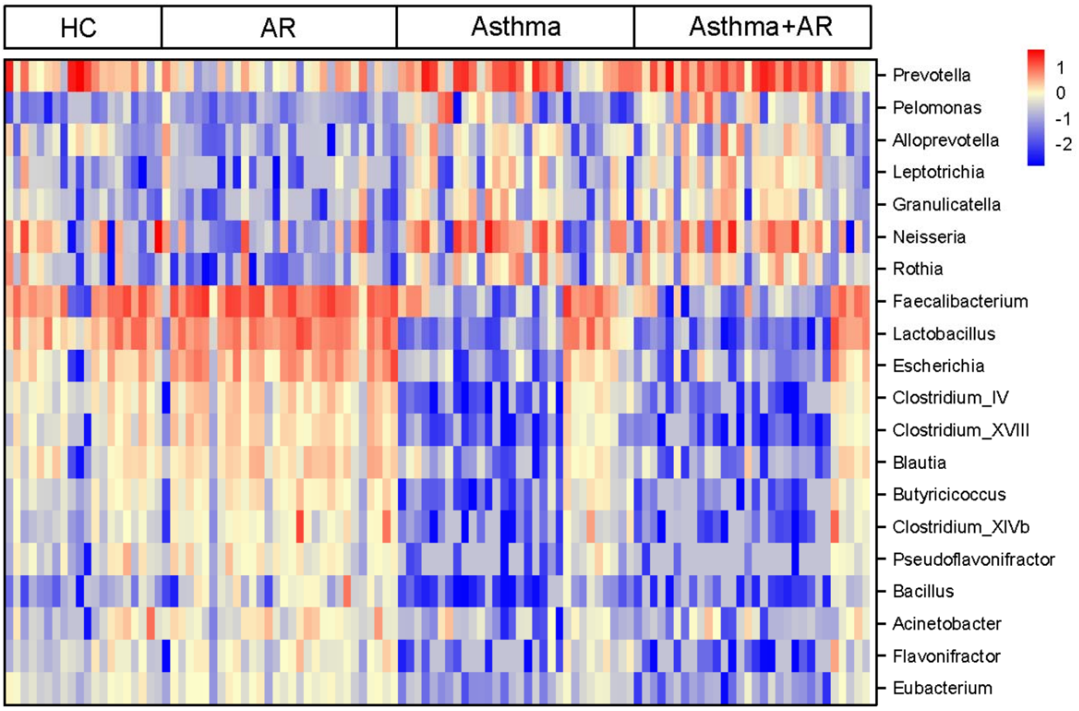

Supplementary Figure 6

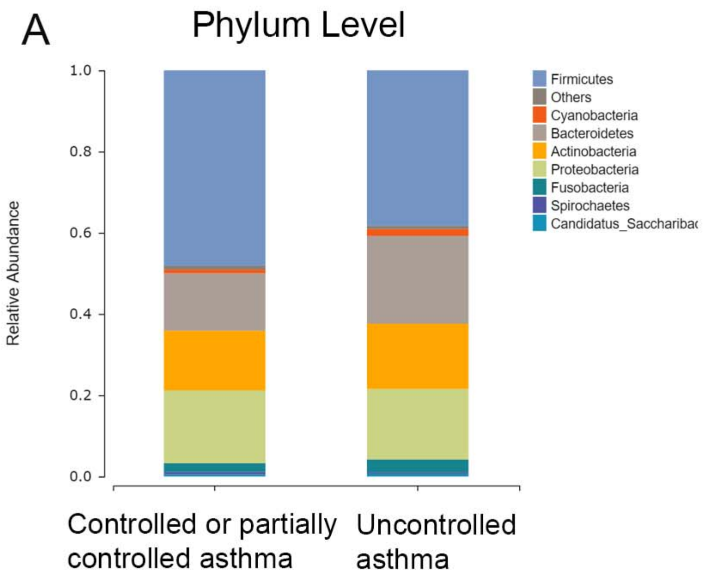

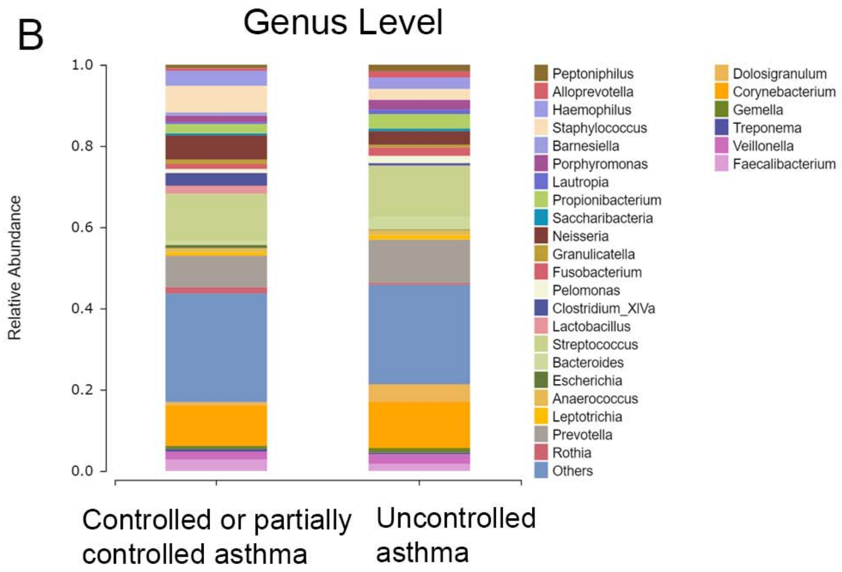

Supplementary Figure 7

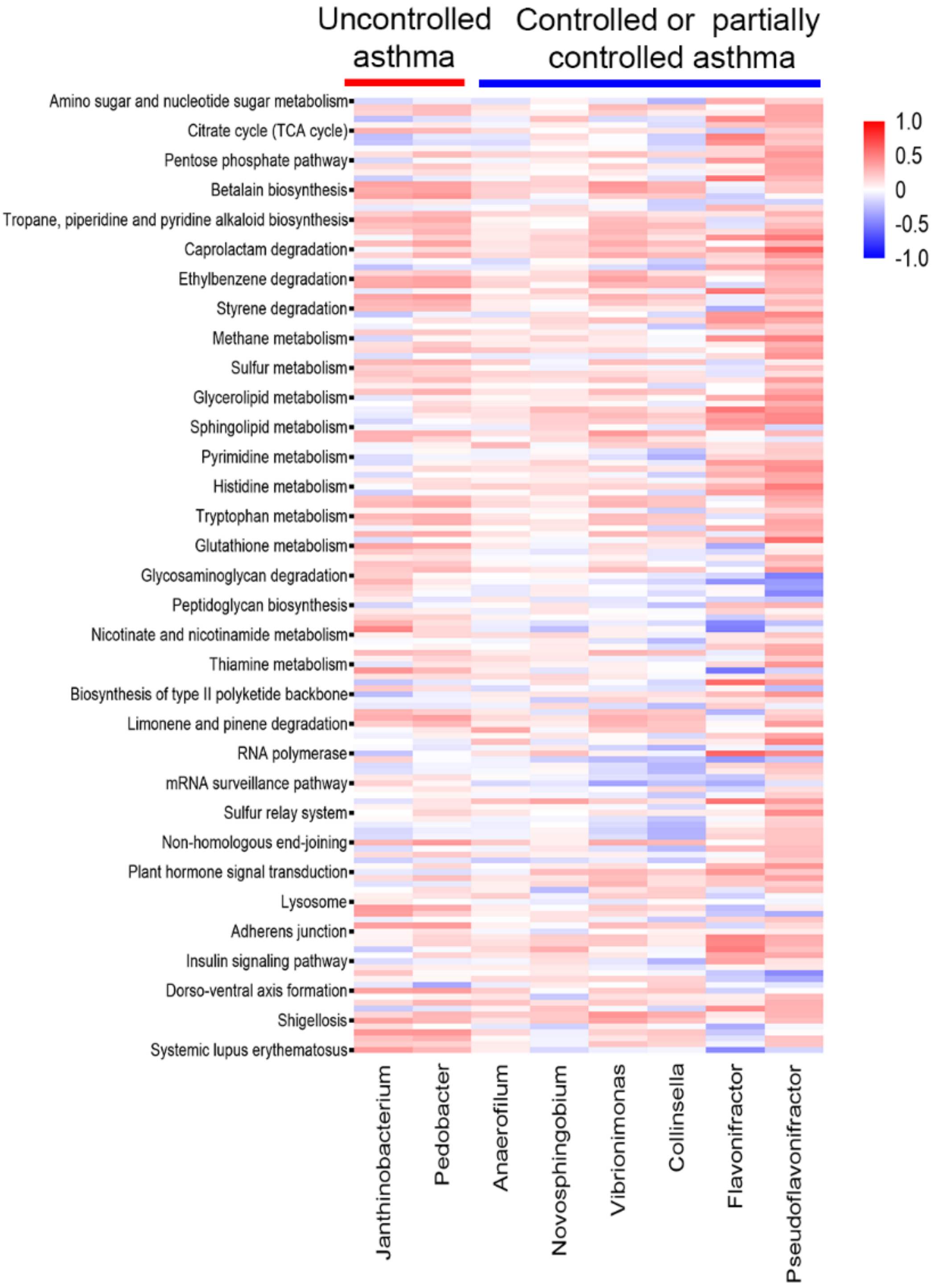

Supplementary Figure 8

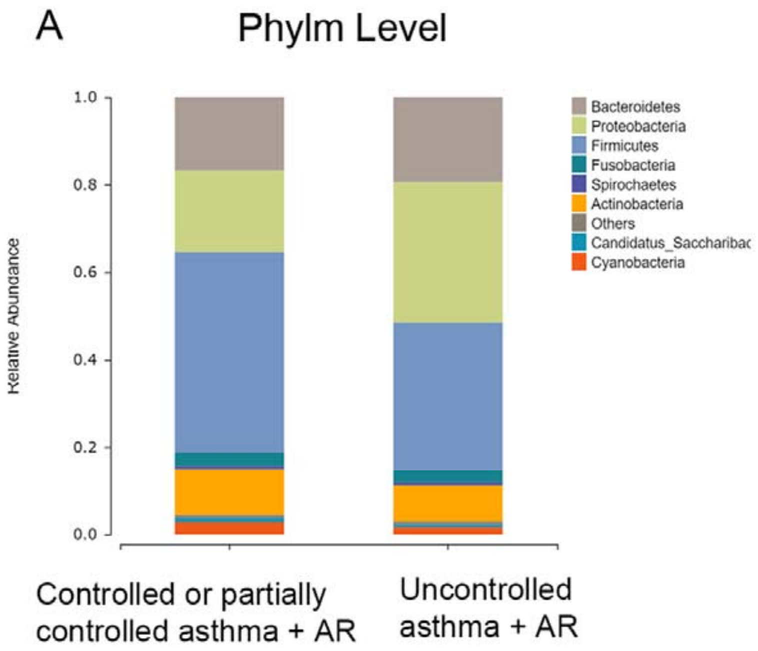

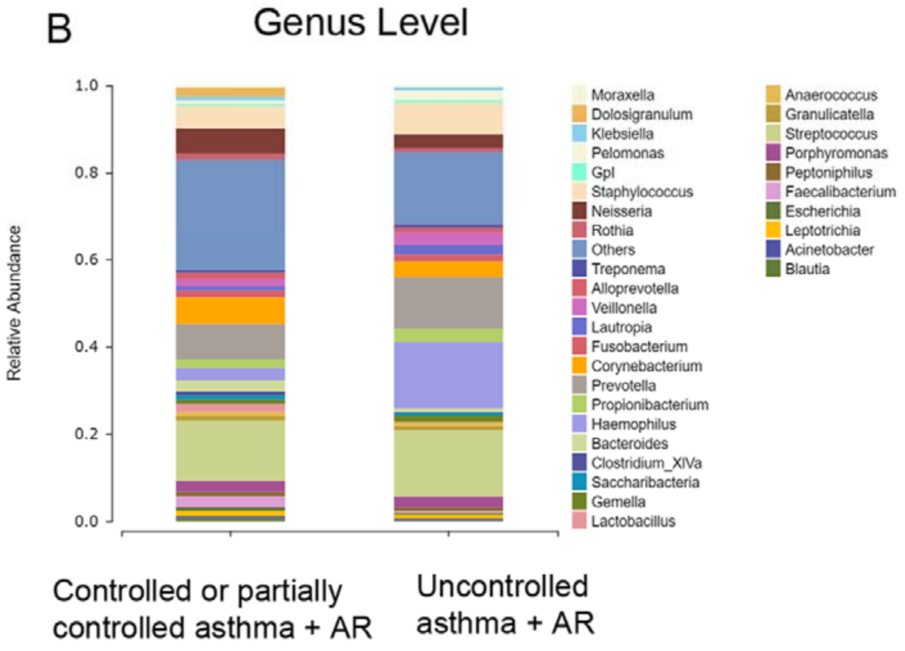

Supplementary Figure 9

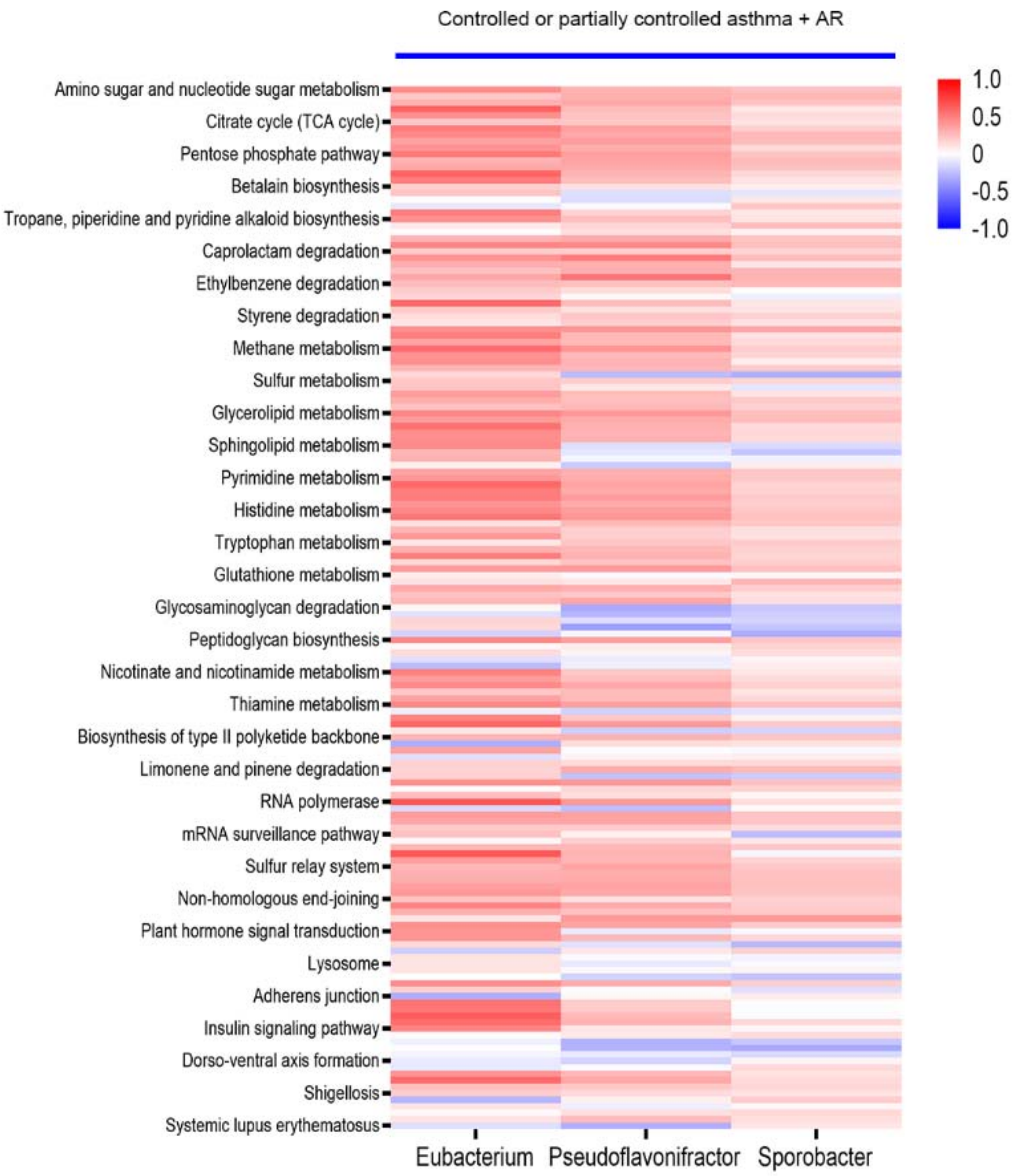

SUPPLEMENTARY TABLES

Supplementary Table 1. Family History and Allergic of Study Participants

**Characteristic**	**HC**	**Patients with allergic disease**				***P* Value**	
		**Total**	**AR**	**Asthma**	**Asthma+AR**	***P*-HC *vs.* Patients**	***P-AR vs. Asthma vs. Asthma + AR***
First degree relatives with history of AR or asthma, n (%)						0.038	0.043
Yes	2 (10.00)	30 (33.33)	11 (36.67)	5 (16.67)	14 (46.67)		
NO	18 (90.00)	60 (66.67)	19 (63.33)	25 (83.33)	16 (53.33)		
Allergic, n (%)							
Grass pollen	0	7 (7.78)	2 (6.67)	2 (6.67)	3 (10.00)	0.434	0.856
House dust mite	0	20 (22.22)	8 (26.67)	3 (10.00)	9 (30.00)	0.021	0.149
Animal	0	3 (3.33)	0	0	3 (10.00)	0.945	0.104

dander							
Medicine	0	9 (10.00)	2 (6.67)	4 (13.33)	3 (10.00)	0.305	0.905
Foods	0	16 (17.78)	3 (10.00)	7 (23.33)	6 (20.00)	0.040	0.474

HC, healthy controls; AR, allergic rhinitis. BMI, body mass index; Categorical variables were compared by Pearson's chi-square test or Fisher exact test.

Supplementary Table 2: Demographics and Clinical Characteristics of Subjects with Asthma According to Disease Control

**Characteristic**	**Subjects with asthma**		***P* - value**
	**Controlled or partially controlled**	**Uncontrolled**	
Subjects (no.)	23 (76.67)	7 (23.33)	
Age (y)	41.04 ± 14.37	41.86 ± 12.38	0.694
Sex ratio (M/F)	11/12	5/2	0.399
Smoking status, %			0.031
Currrent	1 (4.35)	2 (28.57)	
Ex-smoker	0	1 (14.29)	
Never	22 (95.65)	4 (57.14)	
BMI (kg/m^2)	22.33 ± 2.91	23.45 ±4.71	0.902
Atopic, n	12 (52.13)	2 (28.57)	0.399
Medication use, %			
Inhaled corticosteroid	13 (56.52)	1 (14.29)	0.086
Inhaled bronchodilator	12 (52.17)	1 (14.29)	0.104
Leukotriene antagonists	8 (34.78)	1 (14.29)	0.393
FEV_1 % predicted	89.05 ± 13.97	63.53 ±18.88	0.004
FEV_1/FVC %	80.79 ±7.71	65.82 ± 6.97	< 0.001
PEF %	96.40 ± 18.59	63.49 ±23.74	0.003
MMEF75/25 %	66.70 ± 29.32	30.03 ± 12.13	0.001
AQLQ Score	6.54 ± 0.32	6.33 ±0.32	0.105

BMI, body mass index; M, male; F, female; FEV1, forced expiratory volume in first second; FVC, forced vital capacity; PEF, peak expiratory flow; MMEF75/25, maximal mid-expiratory flow; AQLQ, the Asthma Quality of Life Questionnaire.

Supplementary Table 3: Demographics and Clinical Characteristics of Subjects with Asthma and Comorbid AR According to Disease Control

Characteristic	Subjects with asthma + AR		***P* - valiue**
	**Controlled or partially controlled**	**Uncontrolled**	
**Subjects (no.)**	21 (70)	9 (30)	
**Age (y)**	39.43 ± 12.20	37.56 ± 11.76	0.651
**Sex ratio (M/F)**	10/11	3/6	0.691
**Smoking status, %**			0.821
**Currrent**	1 (4.76)	1 (11.11)	
**Ex-smoker**	4 (19.05)	2 (22.22)	
**Never**	16 (76.19)	6 (66.67)	
**BMI (kg/m^2)**	23.97 ± 4.02	23.64 ± 3.81	0.874
**FEV_1 % predicted**	89.01 ± 18.11	65.87 ± 25.19	0.020
**FEV_1/FVC %**	79.71 ± 11.20	69.49 ± 14.82	0.074
**PEF %**	91.44 ± 19.09	69.89 ± 28.31	0.049
**MMEF75/25 %**	68.86 ± 33.13	43.56 ± 25.11	0.071
**AQLQ Score**	6.25 ± 0.51	5.77 ± 0.53	0.017

Values are mean ± SD or n (%). BMI, body mass index; M, male; F, female; FEV1, forced expiratory volume in first second; FVC, forced vital capacity; PEF, peak expiratory flow; MMEF75/25, maximal mid-expiratory flow; AQLQ, the Asthma Quality of Life Questionnaire.

Supplementary Table 4: Relative abundance of dominated bacterial phyla in nasal lavage fluid in patients with AR, asthma, asthma and comorbid AR and healthy controls

Phylum	Mean (AR)	Mean (Asthma)	Mean (Asthma+AR)	Mean (HC)	*P*-value*	FDR[†]
Actinobacteria	9.03	14.97	9.80	14.29	0.157	0.294
Bacteroidetes	5.61	16.25	17.88	15.89	< 0.001	< 0.001
Cyanobacteria	0.30	1.18	2.48[#]	0.31	< 0.001	0.001
Firmicutes	68.73[#]	45.37	41.97[#]	53.55	< 0.001	< 0.001
Fusobacteria	0.63	2.46	3.16[#]	0.92	< 0.001	< 0.001
Proteobacteria	14.44	17.73	22.36	13.46	0.038	0.088

Values are mean. HC, healthy control; AR, allergic rhinitis; Asthma + AR, asthma and comorbid allergic rhinitis. *P-values calculated using the Kruskal-Wallis test; † FDR obtained using the Benjamini-Hochberg procedure. #FDR significant for the comparison of allergic disease group *vs*. HC.

Supplementary Table 5: Relative abundance comparisons at genus level in nasal lavage fluid in patients with AR, asthma, asthma and comorbid AR and healthy controls

Genus	Mean (AR)	Mean (Asthma)	Mean (Asthma+AR)	Mean (HC)	*P* value*	FDR†
Streptococcus	2.98	12.12#	14.65#	2.88	< 0.001	< 0.001
Corynebacterium	5.72	10.16	5.30	10.53	0.162	0.400
Prevotella	1.63#	8.73	9.47	7.88	< 0.001	< 0.001
Faecalibacterium	9.96#	2.46#	1.72#	5.44	< 0.001	< 0.001
Staphylococcus	4.30	5.57	5.82	2.88	0.893	0.921
Neisseria	1.51	5.40	4.96	4.63	0.001	0.008
Lactobacillus	5.83#	1.43#	1.15#	3.78	< 0.001	< 0.001
Haemophilus	0.86	3.50	6.13#	1.42	< 0.001	< 0.001
Dolosigranulum	1.86	1.69	1.36	4.80	0.248	0.513
Clostridium_XlVa	2.01	2.35#	0.56#	4.65	< 0.001	< 0.001
Propionibacterium	1.80	2.63	2.32	1.64	0.928	0.944
Veillonella	0.42	2.09	2.11#	0.75	< 0.001	0.002
Bacteroides	1.09	1.34	1.95	1.06	0.334	0.569
Fusobacterium	0.41	1.47	1.66#	0.52	< 0.001	< 0.001
Lautropia	0.32	0.62	1.36#	0.19	< 0.001	0.005
Alloprevotella	0.30#	0.89	1.35	0.65	0.002	0.013
Rothia	0.33#	1.35	1.28	0.89	< 0.001	0.002
Pelomonas	0.13#	1.17#	1.26#	0.09	< 0.001	0.003
Leptotrichia	0.22#	0.88#	1.17#	0.34	< 0.001	< 0.001
Gemella	0.18	0.93	1.15#	0.27	< 0.001	< 0.001
Granulicatella	0.25	1.00	1.03#	0.29	< 0.001	< 0.001

Values displayed by mean. **P*-values calculated using the Kruskal-Wallis test; † FDR obtained using the Benjamini-Hochberg procedure. #FDR significant for the comparison of

allergic disease group *vs.* HC.

Supplementary Table 6: Relative abundance comparisons at phylum level in Asthma According to Disease Control

**Phylum**	**Uncontrolled asthma (%)**	**Controlled or partially controlled asthma (%)**	***P* value**	**FDR**
Firmicutes	38.22	47.61	0.170	0.628
Bacteroidetes	21.84	14.51	0.202	0.628
Cyanobacteria	1.68	1.03	0.039	0.464
SR1	0.47	0.10	0.045	0.464
Synergistetes	0.01	0.06	0.126	0.589
Acidobacteria	0	0.02	0.224	0.628
Values displayed by mean. † FDR obtained using the Benjamini-Hochberg procedure.				

Supplementary Table 7: Relative abundance comparisons at genus level in Asthma According to Disease Control

**Genus**	**Uncontrolled asthma (%)**	**Controlled or partially controlled asthma (%)**	***P* value**	**FDR**
Propionibacterium	3.52	2.34	0.220	0.797
Pelomonas	1.70	1.01	0.056	0.547
Lautropia	1.16	0.46	0.154	0.717
Campylobacter	0.74	0.32	0.280	0.797
Lachnospiracea_incertae_sedis	0.69	0.14	0.202	0.797
Aquabacterium	0.67	0.37	0.141	0.707
Lachnospira	0.62	0.06	0.297	0.797
Capnocytophaga	0.61	0.32	0.239	0.797
Chryseobacterium	0.51	0.22	0.039*	0.547
SR1	0.47	0.10	0.045*	0.547
Stenotrophomonas	0.39	0.24	0.062	0.547
Pedobacter	0.36	0.21	0.021	0.502
GpI	0.35	0.26	0.030	0.547
Bradyrhizobium	0.31	0.17	0.220	0.797
Oribacterium	0.28	0.12	0.102	0.620

Acidovorax	0.26	0.15	0.070	0.547
Paraprevotella	0.26	0	0.293	0.797
Herbaspirillum	0.25	0.18	0.061	0.547
Values displayed by mean. † FDR obtained using the Benjamini-Hochberg procedure.				

Supplementary Table 8: Relative abundance comparisons at phylum level in Asthma comorbid AR According to Disease Control

Phylum	Controlled or partially controlled asthma + AR (%)	Uncontrolled asthma + AR (%)	*P* value	FDR
Firmicutes	45.24	34.51	0.148	0.754
Proteobacteria	18.55	31.22	0.046	0.754
Verrucomicrobia	0.12	0.08	0.188	0.754
Deferribacteres	0.02	7.39E-4	0.087	0.754
Chloroflexi	0.01	1.63E-3	0.293	0.781
Nitrospirae	1.18E-3	0.00	0.259	0.776
Parcubacteria	6.32E-4	2.56E-3	0.164	0.754
Chlamydiae	0.0	4.99E-4	0.146	0.754

AR, allergic rhinitis. Values displayed by mean. † FDR obtained using the Benjamini-Hochberg procedure.

Supplementary Table 9: Relative abundance comparisons at genus level in Asthma comorbid AR According to Disease Control

Genus	Controlled or partially controlled asthma + AR (%)	Uncontrolled asthma + AR (%)	P value	FDR
Prevotella	8.38	11.97	0.161	0.753
Haemophilus	2.95	13.53	0.161	0.753
Faecalibacterium	2.30	0.33	0.258	0.753
Dolosigranulum	1.94	0.01	0.219	0.753
Lactobacillus	1.60	0.05	0.277	0.753
Rothia	1.43	0.93	0.239	0.753
Clostridium_XlVa	0.87	0.11	0.277	0.753
Pelomonas	0.81	2.30	0.113	0.753
Blautia	0.63	0.13	0.124	0.753
Clostridium_XlVb	0.61	0.01	0.293	0.753
GpI	0.60	0.60	0.174	0.753
Aggregatibacter	0.36	0.11	0.256	0.753
Aquabacterium	0.33	0.78	0.205	0.753
Clostridium_IV	0.28	0.01	0.090	0.753

Butyricicoccus	0.27	0.01	0.222	0.753
Chryseobacterium	0.24	0.49	0.222	0.753
Stenotrophomonas	0.23	0.57	0.161	0.753
Megamonas	0.22	0.02	0.167	0.753
Fusicatenibacter	0.20	0.04	0.103	0.753
Pedobacter	0.20	0.48	0.077	0.753
Eubacterium	0.18	0.07	0.057	0.753
Clostridium_XVIII	0.17	0.02	0.222	0.753
Pseudoflavonifractor	0.17	0.00	0.026	0.753
Bradyrhizobium	0.16	0.32	0.148	0.753
Acidovorax	0.14	0.34	0.175	0.753
Anaerostipes	0.12	0.04	0.161	0.753
Peptostreptococcus	0.12	0.15	0.239	0.753
Herbaspirillum	0.12	0.28	0.175	0.753
Lachnospira	0.11	0.02	0.233	0.753
Ruminococcus2	0.11	0.04	0.230	0.753
Megasphaera	0.10	0.07	0.078	0.753
Faecalicoccus	0.10	0.01	0.226	0.753
Janthinobacterium	0.08	0.25	0.069	0.753
Coprococcus	0.07	0.02	0.203	0.753
Phascolarctobacterium	0.07	0.03	0.233	0.753
Vampirovibrio	0.06	0.00	0.106	0.753

Vibrionimonas	0.06	0.00	0.125	0.753
Collinsella	0.06	0.00	0.086	0.753
Caulobacter	0.05	0.12	0.124	0.753
Gemmiger	0.05	0.01	0.128	0.753
Sporobacter	0.04	0.00	0.038	0.753
Mycoplasma	0.04	0.29	0.134	0.753
Mitsuaria	0.04	0.09	0.069	0.753
Aeromonas	0.03	0.10	0.158	0.753
Brucella	0.03	0.08	0.108	0.753
Citricoccus	0.03	0.06	0.238	0.753
Desulfovibrio	0.03	0.03	0.263	0.753
Fretibacterium	0.03	0.04	0.150	0.753
Rhodococcus	0.03	0.00	0.084	0.753
Microbacterium	0.03	0.06	0.160	0.753
Intestinimonas	0.03	0.00	0.010	0.753
Deinococcus	0.02	0.01	0.238	0.753
Serratia	0.02	0.00	0.181	0.753
Weissella	0.02	0.00	0.271	0.753

AR, allergic rhinitis. Values displayed by mean. † FDR obtained using the Benjamini-Hochberg procedure.
