# Supplementary figures and images for: Nasal Bacterial Microbiome Differs Between Healthy Controls and Those With Asthma and Allergic Rhinitis
Source: Front Cell Infect Microbiol. 2022 Mar 3;12:841995. doi: 10.3389/fcimb.2022.841995 (PMC8928226; doi:10.3389/fcimb.2022.841995)

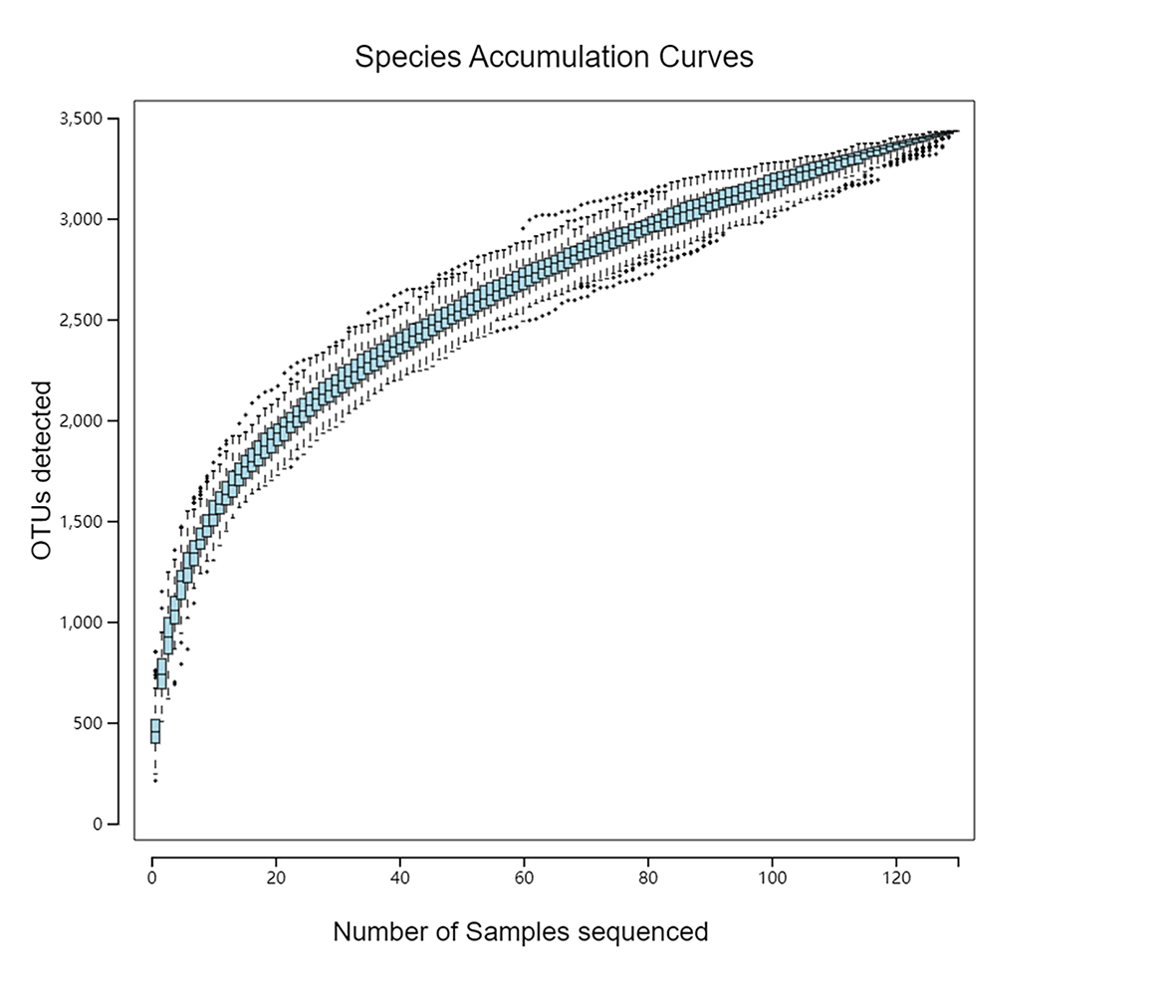

Supplement: Supplementary file 2 [file DataSheet_1.zip › Supplement File1/species accumulation curves.tif]
